# Developing well-calibrated illness severity scores for decision support in the critically ill

**DOI:** 10.1038/s41746-019-0153-6

**Published:** 2019-08-15

**Authors:** Christopher V. Cosgriff, Leo Anthony Celi, Stephanie Ko, Tejas Sundaresan, Miguel Ángel Armengol de la Hoz, Aaron Russell Kaufman, David J. Stone, Omar Badawi, Rodrigo Octavio Deliberato

**Affiliations:** 1 MIT Critical Data, Laboratory for Computational Physiology, Harvard-MIT Health Sciences & Technology, Massachusetts Institute of Technology, Cambridge, MA 02139 USA; 2 Department of Medicine, Hospital of the University of Pennsylvania, Philadelphia, PA 19104 USA; 3 Division of Pulmonary Critical Care and Sleep Medicine, Beth Israel Deaconess Medical Center, Boston, MA 02215 USA; 4 Department of Medicine, National University Health Systems, Singapore, Singapore; 5 Computer Science and Artificial Intelligence Laboratory, Massachusetts Institute of Technology, Cambridge, MA 02139 USA; 6 Division of Clinical Informatics, Department of Medicine, Beth Israel Deaconess Medical Center, Boston, MA 02215 USA; 7 Harvard Medical School, Boston, MA 02115 USA; 8 Biomedical Engineering and Telemedicine Group, Biomedical Technology Centre CTB, ETSI Telecomunicación, Universidad Politécnica de Madrid, Madrid, 28040 Spain; 9 Department of Government, Harvard University, Cambridge, MA 02138 USA; 10 Departments of Anesthesiology and Neurosurgery, University of Virginia School of Medicine, Charlottesville, VA 22908 USA; 11 Department of eICU Research and Development, Philips Healthcare, Baltimore, MD 21202 USA; 12 Big Data Department, Hospital Israelita Albert Einstein, São Paulo, Brazil; 13 Critical Care Department, Hospital Israelita Albert Einstein, São Paulo, Brazil

**Keywords:** Health care, Medical research, Prognosis

## Abstract

Illness severity scores are regularly employed for quality improvement and benchmarking in the intensive care unit, but poor generalization performance, particularly with respect to probability calibration, has limited their use for decision support. These models tend to perform worse in patients at a high risk for mortality. We hypothesized that a sequential modeling approach wherein an initial regression model assigns risk and all patients deemed *high risk* then have their risk quantified by a second, high-risk-specific, regression model would result in a model with superior calibration across the risk spectrum. We compared this approach to a logistic regression model and a sophisticated machine learning approach, the gradient boosting machine. The sequential approach did not have an effect on the receiver operating characteristic curve or the precision-recall curve but resulted in improved reliability curves. The gradient boosting machine achieved a small improvement in discrimination performance and was similarly calibrated to the sequential models.

## Introduction

Illness severity scores are regularly employed for quality improvement and benchmarking in the intensive care unit (ICU) and as covariates for risk adjustment in observational research of critically ill patient cohorts.^1,2^ Common models for estimating severity of illness scores include the Acute Physiology and Chronic Health Evaluation (APACHE), the Simplified Acute Physiology Score, and the Mortality Probability Model.^3–6^ Poor generalization and inadequate model calibration have limited the use of these models for clinical decision support.^7–9^ As medical data become progressively digitized and hence readily analyzable, there is increased interest in, and potential for, the use of this data to develop useful clinical decision support tools.^10^ As accurate quantification of patient mortality risk may inform goals of care discussions and resource allocation, it is an area of clinical predictive modeling that is ripe for improvement.

One of the key challenges has been developing models that are well calibrated.^11–13^ Although prior models have often achieved acceptable observed-to-predicted mortality ratios (OPRs), they frequently under- or over-predict risk across the risk spectrum; in particular, the models tend to be most poorly calibrated in the sickest patients. Critically ill patients represent a heterogeneous risk population with only a minority (about 1/3) of patients constituting what may be defined as high mortality risk, i.e., ≥10%.^14^ The effect of this heterogeneity on predictive models may be best understood in the context of the bias–variance tradeoff.

Although logistic regression allows for the modeling of non-linearities (e.g., polynomial terms, restricted cubic splines) and the manual specification of interactions, complex interactions have been overlooked in the development of prior severity score models. Therefore, the contribution of a feature, for example, the worst heart rate over the first 24 h of hospitalization, to the risk of mortality is considered equal and constant across the spectrum of illness severity. While ensuring less variance and simplifying the models, intuitively, this also results in the creation of a biased model of the underlying data generating process.

More sophisticated machine learning approaches do not require as much input from the modeler and automatically learn relationships directly from the data. This extra flexibility allows these approaches to model non-linearity and feature interactions at the cost of higher variance. Excitement over the power and promise of these methods has continued to swell, but recent work from Christodoulo et al. has challenged the usefulness of machine learning in the development of clinical prediction models.^15^ Their review did not consider logistic regression, penalized or otherwise, technically to be a form of machine learning and did not consider perceptual tasks where the performance of deep learning is uncontested. However, their critique of the state of machine learning in the development of clinical prediction models remains apt.

We hypothesized that, while high-risk patients in ICU cohorts represent a distinct and identifiable group of patients, prior severity scores have been built with methods that are too simple and inflexible to learn to forecast mortality accurately across such heterogeneous groups. While the aforementioned sophisticated machine learning approaches may offer the flexibility to automatically overcome this, these data-hungry methods can be bested by aptly including domain knowledge, especially when data are limited. Because we hypothesized that the model simply needed to treat high-risk patients differently, we sought to explore a sequential modeling approach wherein, after classification of patients as *high risk* with an initial regression model, a *high-risk*-specific regression model is then applied. We explored this approach, as well as a sophisticated machine learning approach, in a large, publicly available, multicenter critical care database.

As the electronic health record (EHR) has given rise to an unprecedented abundance of highly granular clinical data, we expect care centers to increasingly craft bespoke models for severity scoring as well as clinical decision support. With the known shortcomings of previous approaches to severity score development, we hope to inform future model development strategies so as to ensure accurate risk quantification across the board in critically ill patients.

## Results

### Cohort overview

**Fig. 1:**
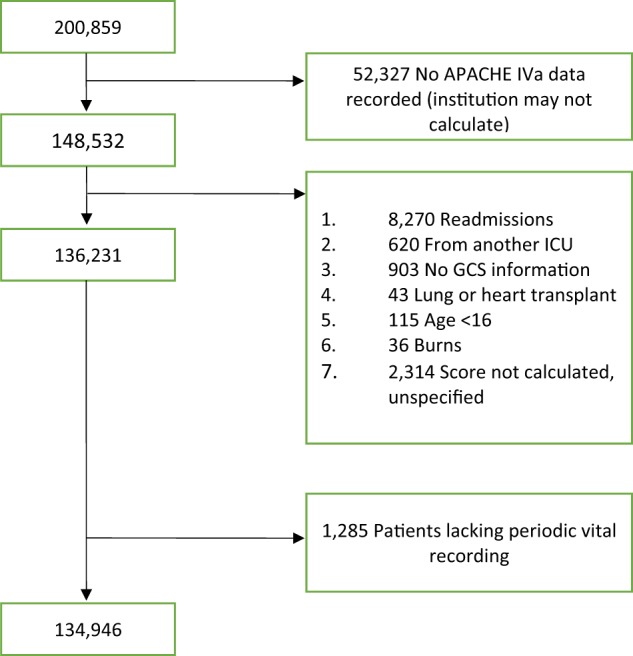
Fluxogram. Cohort selection process

Of the 200,859 ICU stays in the Phillips eICU Collaborative Research Database (eICU-CRD), 148,532 had the required APACHE IVa variables recorded, and 136,231 had the required APACHE IVa variables and also met criteria for the calculation of an APACHE IVa mortality prediction. After excluding patients who lacked at least one recording of automatically captured vital sign data, l34,946 ICU stays remained. The cohort selection is summarized in Fig. 1. Demographic variables and a subset of the features are summarized in Table 1. After randomly splitting off 25% of the initial cohort to form a validation cohort, 101,167 patients remained for model training. All model evaluation metrics are reported on the validation cohort, composed of the held out 33,779 patients who were never used in model training.

**Table 1:** General overview of the cohort features

Variable	Summary measure
Age	63.12 (17.32)
Gender Prop.	54.03% (72,875)
Caucasian ethnicity	77.08% (103,971)
Sepsis	13.32% (17,970)
Cardiac arrest	6.72% (9066)
GI bleed	5.36% (7230)
CVA	7.13% (9616)
Trauma	4.28% (5780)
Unit type: MICU	8.56% (11,545)
Unit type: SICU	6.42% (8664)
Unit type: mixed	55.18% (74,434)
Unit type: other	29.84% (40,247)
Mean HR	84.29 (16.42)
Mean MAP	77.83 (10.14)
Mean RR	19.34 (4.74)
Mean SpO_2_	97.02 (95.5, 98.42)
GCS motor	6 (6.0, 6.0)
GCS eyes	4 (3.0, 4.0)
GCS verbal	5 (4.0, 5.0)
Antibiotics	26.34% (29,785)
Vasopressors	12.06% (13,635)
Ventilatory support	32.96% (44,456)
APACHE IVa mortality probability	0.05 (0.02, 0.13)
Hospital mortality	8.84% (11,925)

*APACHE* Acute Physiology and Chronic Health Evaluation, *CVA* cerebrovascular accident, *GCS* Glasgow coma scale, *GI* gastrointestinal, *HR* heart rate, *MAP* mean arterial pressure, *MICU* medical intensive care unit, *RR* respiratory rate, *SICU* surgical intensive care unit

### Linear models

**Fig. 2:**
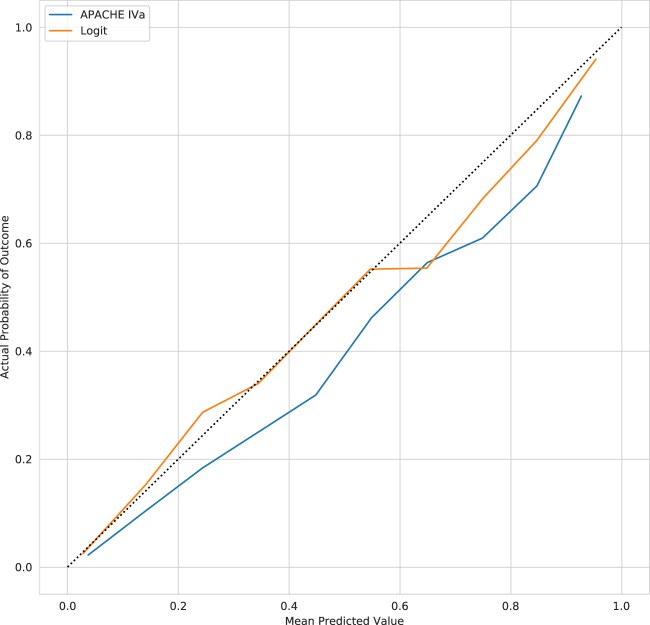
Reliability curves for the APACHE IVa and Logit models

**Fig. 3:**
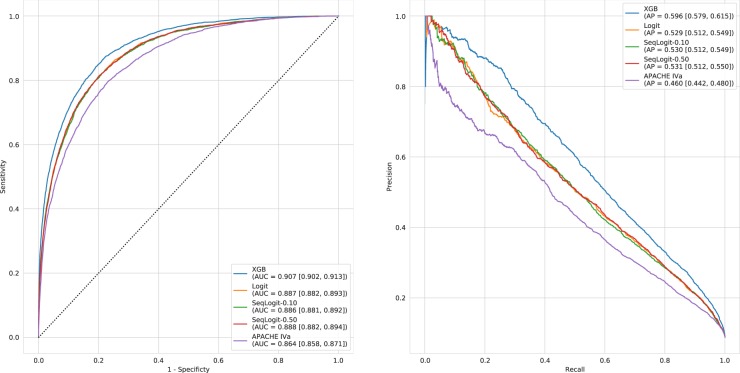
Receiver operating characteristic and precision-recall curves. Receiver operating characteristic and precision-recall curves for all the models. Area under the receiver operating characteristic curve (AUC) and average precision (AP) are provided for each model along with 95% confidence intervals obtained from bootstrapping

Reliability curves for the APACHE IVa model and our logistic regression model (Logit) applied to our validation cohort are shown in Fig. 2. The older APACHE IVa model systematically over-predicts mortality across the risk spectrum. The degradation of model calibration in models like APACHE has been previously attributed to changes in treatment efficacy over time, cultural changes in propensity to forgo care, ICU utilization, and frequency of early discharge.^16^ However, other work examining model degradation attributed the phenomenon to changes in case mix rather than changes in predictor outcome relationships or outcome frequency.^17^ The Logit model appears better calibrated in patients on the lower end of the predicted risk spectrum but becomes less well calibrated as the predicted risk of mortality increases. Despite this evident miscalibration, the OPR for the Logit model is 0.99 [0.96, 1.02] as compared to 0.74 [0.72, 0.76] for the APACHE IVa model. The Logit model also achieved superior discrimination with an area under the receiver operating characteristic curve (AUC) of 0.887 [0.882, 0.893] as compared to an AUC of 0.864 [0.858, 0.871] for the APACHE IVa model. Similarly, the Logit model had an average precision (AP) of 0.529 [0.512, 0.549] as compared to an AP of 0.460 [0.442, 0.480] for the APACHE IVa model. Figure 3 displays the receiver operating characteristic curves and precision-recall curves in the held-out validation cohort for these models, as well as all models trained in this study.

### Sequential models

**Fig. 4:**
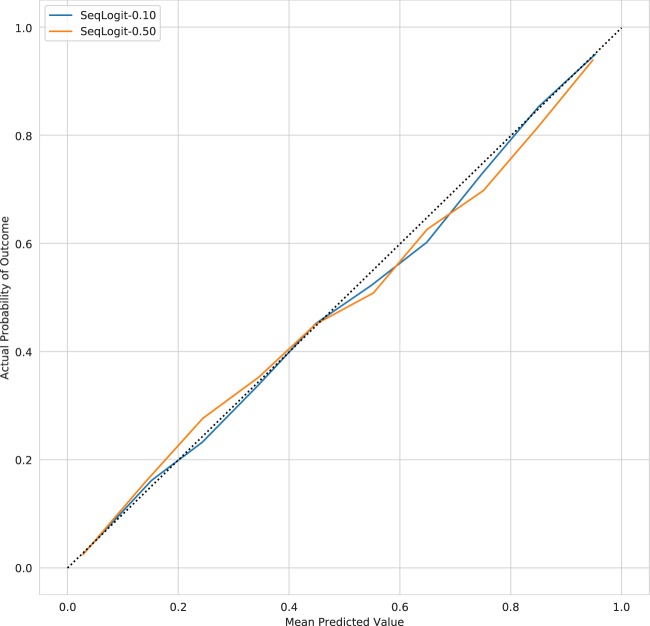
Reliability curves for sequential models

The OPR for SeqLogit-0.10 and SeqLogit-0.50 in the validation cohort are 0.98 [0.95, 1.01] and 1.00 [0.97, 1.03], respectively, indicating similar overall calibration performance, but the reliability curves for these models shown in Fig. 4 suggest that both sequential models are better calibrated than the baseline Logit model alone at the higher end of the risk spectrum. As is evident in Fig. 3, the AUC and AP of the sequential models is equivalent to the Logit model. Inspection of the feature importance of the Logit model and the high-risk components of the sequential models provides support for the general intuition of sequential approach. For example, in the Logit model, *age* was the most important feature with a coefficient of 0.52 on the log odds scale; it drops to fifth most important feature in the high-risk component of the SeqLogit-0.10 model with a coefficient of 0.38 and eighth most important in the high-risk component of the SeqLogit-0.50 model with a coefficient of 0.32. The different contribution of these features can be understood in terms of the change in distribution of the feature in data on which the model is being trained as case mix necessarily shifts. The mean age in the training cohort was 63 years with a standard deviation of 17.30, but when we examine the high-risk cohort identified by the first step of the SeqLogit-0.10 model, we see the mean age increase to 71 years with a decreased standard deviation of 14.78. In the higher-risk subset, age is higher, but with lower variability, and thus less informative for prediction. Similarly, the maximum lactate level on the first day was a top 10 feature in the high-risk component of the SeqLogit-0.50 model with a coefficient of 0.37 but only a coefficient of 0.16 in the Logit model. The intuition is similar to that of age but in reverse. In the whole training cohort, we expect a wide distribution of lactate and a normal to slightly elevated average lactate, but in the higher-risk patients we expect a higher average lactate and a broader spread. In fact, the mean of the maximum lactate on day 1 for patients in the whole training cohort was 3.11 with a standard deviation of 3.17, and this same feature had a mean of 8.44 with a standard deviation of 5.90 in patients stratified as high risk in the first step of the SeqLogit-0.50 model. The coefficients for each feature of the Logit model and the high-risk components of the sequential models are provided in full in Supplemental Materials.

### Gradient boosting machine

**Fig. 5:**
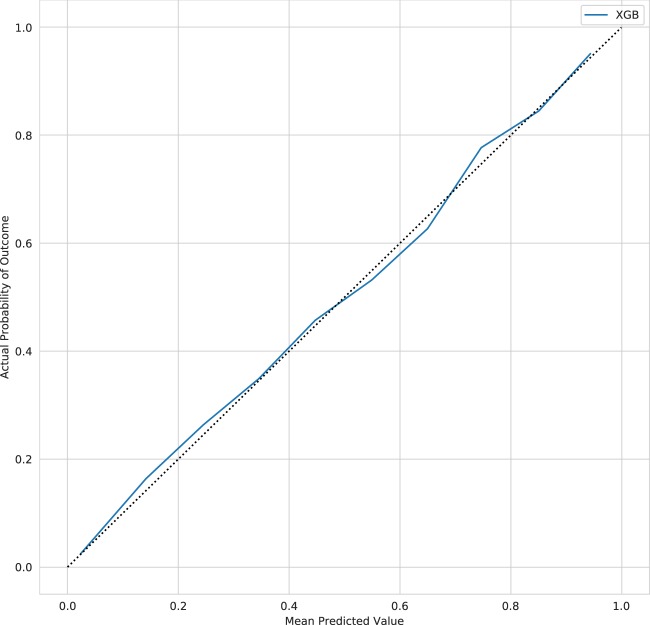
Reliability curves for the extreme gradient boosting model

A subset of the hyperparameters for the xgBoost (extreme gradient boosting (XGB)) model as selected by ten-fold cross-validation (CV) are summarized in Table 2. The best model was chosen based on the optimal negative log-loss and is composed of 1000 trees with a permitted maximum tree depth of 12. The reliability curve for the XGB model is shown in Fig. 5: the model appears well calibrated but varies between slight under- and over-prediction as the predicted risk increases resulting in an overall OPR of 1.04 [1.01, 1.07]. The AUC and AP were slightly superior to all other models in this study at 0.907 [0.902, 0.913] and 0.596 [0.579, 0.615], respectively.

**Table 2:** XGB hyperparameters

Hyperparameter	Value
Learning rate	0.01
Number of trees	1000
Max. tree depth	12
Row sampling	0.6
Column sampling	0.75

The XGB model hyperparameters as selected by ten-fold cross-validation

*XGB* extreme gradient boosting

## Discussion

This work explores strategies for improving the calibration of severity of illness models. We hypothesized that existing models are too inflexible to adequately model the risk of mortality across the full spectrum of illness severity. Furthermore, we postulated that, at minimum, patients with a high risk of mortality signify a distinct group of patients requiring a separate disease severity model mapping their physiological data to the probability of mortality. To examine this further, we developed an updated and augmented severity score based off the APACHE IVa model using multivariate logistic regression as a baseline and then developed models composed of a combination of two logistic regression models, termed sequential models. The sequential models first used the full cohort regression model to assign risk of mortality, and when that risk exceeds a predefined cutoff, a second model trained in only patients above that cutoff in the training set is then employed to estimate risk. We examined this approach with a cutoff of 0.10 and 0.50 risk of mortality. We compared this simple approach to a sophisticated, state-of-the-art machine learning method: the gradient boosting machine.

Inspection of the reliability curves suggests that the sequential modeling approach results in an improvement in calibration in higher-risk patients over the full cohort model alone, but with no effect on AUC or AP, and minimal effect on the overall OPR. In comparison, the XGB model slightly bests the linear models in terms of AUC, with a larger advantage in terms of AP; the XGB model appears well calibrated on inspection of the reliability curve but varies between under- and over-prediction across the risk spectrum resulting in an over-prediction of overall mortality by 4%. As would be expected, all models improve on APACHE IVa given that they are trained and deployed in data from the same generating source and thus are closer to the validation data both temporally and spatially.

The fundamental premise of this work, that accurate risk quantification with severity of illness models requires that the model be able to quantify risk differently in patients with higher disease severity, is supported by the improved calibration seen on the reliability curves for the sequential models. In agreement with our intuition, features that are important in discerning risk of death across the whole cohort are less important in the highest-risk patients, and the sequential approach allows a re-weighting that supports improved risk quantification at the higher end of the risk spectrum. While the XGB model allows for up to 12 levels of interaction, the gain in discrimination is marginal at the cost of increased variance, with slight overall over-prediction of mortality in the validation cohort; furthermore, this complex model is harder to interpret and developers will need to weigh this difficulty against the small discriminative gains provided.

However, there are two other important conclusions to draw from this work. Foremost, the worst performing model, APACHE IVa, still demonstrated an AUC of 0.864 [0.858, 0.871], not substantially lower than the XGB model that had the highest AUC at 0.907 [0.902, 0.913], and yet it was very poorly calibrated. Shah et al. highlighted this problem in the broader context of clinical machine learning, referring to calibration as the “Achilles’ heel of prediction.” Proper calibration is crucial, especially for informing care decisions, and even strong discrimination performance alone is insufficient for this purpose. That is, while thresholding risk at 50% can assign a label of survival or expiration, for clinical decision support a 10% chance of mortality is not the same as a 40% chance, just as a 95% chance differs from a 55% chance. In addition, with respect to discrimination, the fact that a sophisticated method capable of modeling complex interactions did not substantially outperform less complex methods, or even the out-of-date APACHE IVa model, suggests to us that substantial gains are unlikely to be related to algorithmic advancement alone, at least while we are reliant on a small subset of structured clinical data. While complex methods like xgBoost have led to performance gains in various task domains, allowing models to capture non-linearity and interaction directly from engineered features is less important than the engineering of such features.^18–20^ Better quantification of mortality risk with improved discriminative performance will likely require the identification of other variables that explain more of the variance observed. As a significant amount of patient data reside in unstructured formats like images and clinical notes, modeling approaches (such as deep learning) that can automatically extract novel features from these sources represent an emerging strategy for advancing the current state-of-the-art.^21–23^ However, we emphasize that it is these rich data sources that demonstrate the usefulness of deep learning, and the application of deep learning to structured inputs traditionally used in clinical models has not yielded performance gains. Extending the intuition behind the sequential approach, we not only anticipate that certain features will contribute more strongly in certain subpopulations but also that the incorporation of new features selectively within relevant subgroups will improve predictive performance.

This work has several important limitations. Foremost, because no other publicly available database (including MIMIC-III) provides the variables used to derive the APACHE IVa score and we chose to use this expansive feature set, we were unable to test the sequential models on a truly external validation set as is expected by the Transparent Reporting of a multivariable prediction model for Individual Prognosis Or Diagnosis (TRIPOD) statement.^24^ We have attempted to allay some of this shortcoming by selecting hyperparameters with ten-fold CV, evaluating our final model on a validation set that the model never encountered during training, using data that is publicly available online, and making our code publicly available online.^25^ However, the lack of another publicly available database in which to test our models in full compliance with the TRIPOD statement represents this work’s greatest shortcoming.

Another important limitation of this work lies in the feature engineering. Unlike the APACHE IVa system that uses the worst value for vital sign parameters such as heart rate, the models we developed automatically processed vital sign inputs from streaming 5-min medians and averaged these data over the first 24 h. Values curated to be the worst are not only used in the standard APACHE score calculation and might be more predictive of mortality than an average over the first day but are also more subject to technical (artifacts, monitoring inaccuracies), user input, and data storage and retrieval issues. In this current digital age, perhaps precise definitions need to be modified so “worse” values are better defined in terms of the data collected, which is often stored as a median value of some kind such as ≥5 min time interval medians. In addition, average values may be more representative of the physiological state as sustained over the period of the APACHE day. However, the goal of this work was to compare the sequential approach to the commonly used simple linear modeling approach and a more complex machine learning approach, and this type of feature selection was used in an internally consistent manner across all of these methods. In fact, our model performed as well or better than the standard APACHE worse value method. As such, it is unlikely that it diminishes the conclusion of this paper that sequential modeling improves severity score calibration.

A further limitation is that arbitrary cutoffs for *high risk* of 0.10 and 0.50 were used, and in future work the cutoff point or points should be considered a hyperparameter chosen via a CV procedure in a data-driven manner. Selection bias also represents an important shortcoming: a significant limitation of this study is the number of patients for whom APACHE data were not present and/or an APACHE IVa score was not calculated as they were excluded. It is unlikely that these data were missing completely at random (MCAR), and thus the missingness is likely informative. Therefore, exclusion of these patients may have biased our models and comparisons. While this does not specifically detract from the goal of this study, it is important to note that models developed in this manner would not be applicable to patients who would have been inadvertently excluded by this selection bias, and prospective analysis is crucial to safely developing and deploying these tools.

As healthcare continues to digitize and EHR data continues to proliferate, we expect that hospitals will increasingly craft bespoke models in order to quantify the probability of outcomes relevant to clinical decision-making. In the ICU setting, the quantification of disease severity and the risk of hospital mortality can optimize resource allocation; motivate goals of care conversations; and allow clinicians, patients, and families prognostic insight. Because of the wide spectrum of disease severity present in ICU cohorts, we believe strategies for adequately calibrating models across the risk spectrum are necessary. We demonstrate that a sequential approach that combines two linear models is a valid strategy but provides only a modest improvement, and further work is needed to ultimately deliver models that accurately quantify risk in every ICU patient.

## Methods

### Data source

The eICU Collaborative Research Database (eICU-CRD) is a large, open access, multicenter critical care database made available by Philips Healthcare in partnership with the MIT Laboratory for Computational Physiology.^26^ It holds data associated with 200,859 ICU admission for 139,367 unique patients across the United States; we employed data from 2014 and 2015. Version 2.0 of the database is publicly available at https://eicu-crd.mit.edu/.

### Ethical approval

This study was exempt from institutional review board approval due to the retrospective design, lack of direct patient intervention, and the security schema for which the re-identification risk was certified as meeting safe harbor standards by Privacert (Cambridge, MA) (Health Insurance Portability and Accountability Act Certification no. 1031219-2).

### Cohort feature extraction

Patients were required to have the variables necessary to calculate the APACHE IVa score as well as having a valid APACHE IVa result recorded in the database. By requiring a valid APACHE IVa score, the following exclusion criteria were implicitly imposed: age <16 years, readmissions, ICU transfers, patients with ICU length of stay <4 h, patients with hospital length of stay >365 days, and patients admitted for burns or non-renal, non-hepatic transplant. In addition to these criteria, patients were also required to have at least one recording of each of the following: automated vital sign recording, laboratory result data, and treatment documentation.

The models developed in this study were meant to recapitulate and extend other well-known mortality models. They consist of a combination of the original APACHE IVa features with features engineered from routinely collected EHR vital signs, laboratory test results, diagnoses, and administered treatments.

The APACHE IVa features included were: admission diagnoses, whether thrombolytics were given, whether the patient was actively receiving any of the certain predefined treatments, the components of the Glasgow coma scale, whether or not the patient had an internal mammary graft, whether or not the patient had a myocardial infarction within the past 6 months, and ventilation and intubation indicator variables. The admission diagnoses were grouped into meaningful categories using a special text string for describing a search pattern known as a regular expression. In this approach, the presence or absence of a search string is used to classify the strings into groups.

With respect to the vital sign data, the models we develop incorporate the mean over all values in the first 24 h in the ICU; these mean values were used for the following variables: heart rate, respiratory rate, oxygen saturation, systolic, diastolic, and mean arterial pressure. Although the APACHE IVa model uses the *worst values*, our new models pull data that are derived from automatically captured signals, and these streaming systems tend to have noise; thus the minimum and maximum values may reflect artifact. Although the automatically captured temperature was extracted for each patient, it was missing for a substantial majority and was excluded from the analysis as its fidelity was considered suspect.

For laboratory data, the minimum and maximum values in the first 24 h were initially extracted. For serum bicarbonate, chloride, calcium, magnesium, base excess, platelets, hemoglobin, phosphate, fibrinogen, pH, and hematocrit, the minimum value was selected. For serum creatinine, blood urea nitrogen, bilirubin, lactate, troponin, amylase, lipase, B-natriuretic peptide, creatinine phosphokinase and prothrombin time, the maximum value was selected. For serum sodium and glucose, the most abnormal value was defined as the value with the greatest deviation from the normal range. For white blood cell and neutrophil counts, if any measurements were lower than the lower limit of the normal range, the minimum value was used; if the minimum was within normal range, then the maximum was used.

Treatment information from the first 24 h regarding the administration of vasopressors, antiarrhythmic agents, antibiotics, sedatives, diuretics, and blood products was also extracted and these data were included as binary indicator variables.

### Training and validation split

Following the extraction of the features from the database for all included patients, 25% of the dataset was randomly sampled and held out for derivation of a validation cohort. The remaining 75% of the data were then used to develop the models.

### Modeling procedures

Logistic regression (Logit) was chosen as the base model and for the components of the sequential models as this approach is common in clinical prediction and severity score development, is easily interpretable, and often produces results as good, if not superior to, more complex approaches.^15^ The sequential models were constructed using a cutoff definition of 0.10 risk of mortality and 0.50 risk of mortality; they are titled SeqLogit-0.10 and SeqLogit-0.50, respectively. In these models, the base Logit model, which is fit on data from the entire training set, is used to initially predict the risk of mortality and if that predicted risk is above the cutoff, a second multivariate logistic regression model trained only in patients above the cutoff is used to predict the mortality. When deployed in the validation cohort, the base Logit model first predicts the risk and if the risk is above the cutoff, the second model is used to predict the risk of mortality.

In order to compare the Logit model and sequential models to the current state-of-the-art approach for prediction with structured data, a gradient boosting machine model was implemented. This approach has recently gained traction in clinical predictive modeling with state-of-the-art performance at predicting risk of readmission and risk of acute kidney injury.^19,20^ We utilized the XGB implementation developed by Tianqi Chen.^27^ The gradient boosting approach allows for the development of a strong learner from many weak learners. For the classification task, classification trees of a specified complexity are trained iteratively, each tree being trained on the residual from the linear combination of all prior trees (with the first tree trained on a pseudo-residual). The complexity of the gradient boosting machine is controlled by various factors including the number of trees to be fit, the size of the random sample of the data to be used for each tree, the number of features to be randomly selected for the construction of each tree, the tree depth, and the shrinkage factor (learning rate). These hyperparameters allow for fine control of the model flexibility and thus, if chosen correctly, allow for the development of a model that is hypothetically close to the optima of the bias–variance tradeoff and reduces overfitting.

All hyperparameters were selected via ten-fold CV using only the training data. Because there are many hyperparameters to select with this approach, and because fitting these models is computationally expensive, the CV hyperparameter search was carried using a random search strategy: a grid of possible hyperparameter combinations was randomly sampled from 100 times for a total of 1000 model fits. This approach has been shown to be highly successful and spares substantial wasted CPU time in areas of the hyperparameter space that are far from optimal.

Because of the differences between Logit and XGB, different preprocessing steps were applied depending on which method was being applied. Regression based methods are not capable of dealing with missing data, and samples with any missing values are excluded, that is, missing data are assumed to be MCAR unless adjustment for missingness is performed (e.g., inverse probability weighting to model the mechanism of missingness). To overcome this, the fitting of regression models was carried out using a pipeline that performed multiple imputation using Bayesian Ridge Regression as implemented in the current development version of scikit-learn.^28^ In addition, this pipeline also normalized the data using the mean and standard deviation from the training data. In contrast, tree-based methods are implemented such that they may automatically learn to handle missing data directly from the data, and there is no need for normalization. As such, no preprocessing was performed on the data used to train the XGB models.

No model recalibration procedures were applied to either approach. Logistic regression-based approaches maximize the likelihood estimator and in turn, minimize the *negative log loss* given by:

$$\frac{{ - 1}}{N}{\sum} {y_i{\mathrm{log}}\left( {p_i} \right)} + \left( {1 - y_i} \right){\mathrm{log}}\left( {1 - p_i} \right)$$

Minimization of this loss function directly optimizes the probabilities, *p*_*i*_, produced by the model and therefore logistic regression produces inherently well-calibrated probabilities. Although Niculescu-Mizil et al. have demonstrated that gradient boosting classification tree-based approaches do not produce well-calibrated probabilities, their work specifically examined AdaBoost, a different implementation, which minimizes the squared exponential loss function.^29^ In contrast, XGB can be trained with its objective to minimize the log loss, and therefore, like regression, can produce well-calibrated probabilities without recalibration procedures. Eschewing this step allowed us to use all of the training data to fit the models as re-calibration requires a second held out dataset.

### Model performance evaluation

Calibration was examined graphically with the use of reliability curves; this is recommended as the optimal way to assess calibration, and the Hosmer–Lemeshow test was not performed as it offers minimal insight and can be misleading in large samples.^30^ Although not an optimal measure of calibration across the risk spectrum, the overall OPR was also calculated for each model. Discrimination was examined graphically by plotting receiver operating characteristic curves and quantitatively by calculating the AUC. Precision-recall curves were also generated and AP was also assessed. Uncertainty around AUC, AP, and observed-to-predicted mortality estimates was quantified by the construction of 95% confidence intervals obtained via bootstrapping: 2000 bootstrap samples were used for each interval.

### Statistical analysis

Normality was assessed by examination of variable distribution. Continuous variables are presented as means and standard deviations if normally distributed or medians with 25th and 75th percentiles otherwise. Categorical variables are presented as the percentage of the whole and sum total. As mentioned above, confidence intervals were derived via the construction of 2000 bootstrap samples for estimated metrics and a null-hypothesis testing framework was not applied.

### Software

The data were extracted from the database with the use of structured query language. This work was developed with Python 3.6. The scikit-learn and xgBoost packages were used for model development, and graphics were produced with the use of the matplotlib package.^27,28^

### Reporting summary

Further information on research design is available in the Nature Research Reporting Summary linked to this article.

## Supplementary information

Supplemental Material

Reporting Summary

## Data Availability

The dataset analyzed during the current study is publicly available at https://eicu-crd.mit.edu/.

## Supplementary information

**Supplementary Information** accompanies the paper on the *npj Digital Medicine* website (10.1038/s41746-019-0153-6).

## Code availability

All of the code for this work is publicly available at https://github.com/cosgriffc/seq-severityscore.
