# Supplementary material for: Developing well-calibrated illness severity scores for decision support in the critically ill
Source: NPJ Digit Med. 2019 Aug 15;2:76. doi: 10.1038/s41746-019-0153-6 (PMC6695410; doi:10.1038/s41746-019-0153-6)
Supplement: Supplementary file 1 — Supplemental Material [file 41746_2019_153_MOESM1_ESM.pdf]

Supplemental

Feature	Logit Model	SeqLogit-0.10 (HR component)	SeqLogit-0.50 (HR component)
abx	0.0136	-0.0153	-0.0196
adx_ARF	-0.0485	-0.0658	-0.0340
adx_Asthma-Emphys	-0.0856	-0.1023	-0.0760
adx_CABG	-0.3187	-0.1477	0.0000
adx_CardiacArrest	0.1208	0.1122	0.0551
adx_ChestPainUnknown	-0.0288	-0.0296	-0.0402
adx_CHF	-0.0178	-0.0604	-0.0940
adx_Coma	-0.0370	-0.0376	-0.0713
adx_CVA	0.2561	0.2430	0.0892
adx_CVOther	-0.0731	-0.0516	-0.1290
adx_DKA	-0.3346	-0.0705	-0.0572
adx_GIBleed	-0.1604	-0.1429	-0.1754
adx_GIObstruction	-0.0406	-0.0661	-0.0514
adx_Neuro	-0.1075	-0.0895	-0.0924
adx_Other	-0.1967	-0.1894	-0.2026
adx_Overdose	-0.2818	-0.0673	-0.0571
adx_PNA	0.0059	-0.0423	-0.1195
adx_RespMedOther	-0.0099	-0.0799	-0.2103
adx_Sepsis	-0.0970	-0.1896	-0.3559
adx_Trauma	0.0325	0.0105	-0.0774
adx_ValveDz	-0.2745	-0.1154	-0.0936
age	0.5231	0.3757	0.3159
albumin_min	-0.3409	-0.2682	-0.1973
amylase_max	0.0121	0.0026	0.0113
aniongap_max	0.0207	0.0360	-0.0340
antiarr	-0.0185	-0.0362	-0.0208
apache_ejectfx	0.0112	-0.0099	-0.0083
apache_graftcount	0.0212	-0.0183	0.0000
apache_ima	-0.1441	0.0443	0.0000
apache_intubday1	0.1127	0.1026	0.0606
apache_midur	0.0013	0.0007	0.0423
apache_thrombolytics	0.0144	0.0156	-0.0015
apache_ventday1	0.3577	0.3055	0.1862
baseexcess_min	-0.0377	0.0081	-0.0119
bicarbonate_min	-0.1653	-0.2120	-0.1432
bilirubin_max	0.1159	0.1664	0.0783
blood_product	0.0358	0.0123	-0.0130

bnp_max	0.0295	-0.0049	0.0493
bun_max	0.1409	0.1416	0.0291
calcium_min	0.1003	0.1109	0.1483
chloride_min	-0.2150	-0.1786	-0.2032
cpk_max	-0.0079	0.0040	-0.0470
creatinine_max	-0.0712	-0.0895	-0.1068
dbp_aperiodic_mean	0.2386	0.2375	0.3196
diuretic	-0.0080	-0.0236	-0.0347
eth_asian	0.0053	0.0130	-0.0227
eth_caucasian	0.0271	0.0631	0.1158
eth_hispanic	-0.0007	0.0217	0.0959
eth_native_american	-0.0056	0.0048	-0.0070
eth_other	0.0108	0.0358	-0.0113
fibrinogen_min	-0.0342	-0.0113	0.0146
gcs_eyes	-0.1603	-0.1859	-0.0535
gcs_meds	0.0371	0.0232	-0.0836
gcs_motor	-0.2771	-0.3917	-0.2124
gcs_verbal	0.0017	0.0828	0.0312
glucose	-0.0081	-0.0325	-0.0289
height	-0.0129	-0.0028	-0.0359
hematocrit_min	0.4049	0.3745	0.5833
hemoglobin_min	-0.4948	-0.4161	-0.5786
hr_mean	0.2536	0.2063	0.1281
inr_max	-0.0217	0.0259	0.1515
ioncalcium_min	-0.0001	-0.0231	-0.0770
lactate_max	0.1569	0.1709	0.3680
lipase_max	-0.0111	-0.0353	-0.0342
lym	-0.0634	0.0097	0.1236
magnesium_min	0.0414	0.0463	0.1409
male_gender	0.0659	0.0534	0.0709
map_aperiodic_mean	-0.4397	-0.4293	-0.5419
pao2_min	-0.0215	0.0087	0.0112
ph_min	-0.0089	-0.0989	-0.1012
phosphate_min	0.0664	0.0660	0.0278
platelet_min	-0.1020	-0.0858	-0.1628
pmn	-0.0099	0.0024	0.0267
potassium	-0.0041	-0.0156	0.0200
pressor	0.1288	0.1704	0.1780
pt_max	0.0962	0.0748	-0.0178
ptt_max	0.0346	0.0170	-0.0225

rr_mean	0.2238	0.2214	0.2425
sbp_aperiodic_mean	-0.0511	-0.0240	-0.0837
sedative	-0.0272	-0.0239	-0.0751
sodium	0.0676	0.0643	0.0945
spo2_mean	-0.2690	-0.4341	-0.5485
tropi_max	0.0108	0.0137	-0.0011
tropt_max	0.0005	0.0012	0.0306
unit_Cardiac_ICU	0.0517	0.0758	0.1524
unit_CSICU	-0.0569	-0.0212	0.0701
unit_CTICU	0.0187	-0.0003	0.0717
unit_Med_Surg_ICU	-0.0631	-0.0273	0.0596
unit_MICU	0.0083	0.0290	0.0835
unit_Neuro_ICU	0.0588	0.0602	0.1385
unit_SICU	0.0089	0.0209	0.1199
wbc	0.0506	0.0692	0.0310
weight	-0.0682	-0.0524	-0.0303

The above table provides the coefficients for the logistic model, as well as the high-risk components of the sequential components. It highlights how the high-risk sub-population models weight features differently than models trained on the entire population.
